# Diaphragmatic perforation after transcatheter arterial chemoembolization of hepatocellular carcinoma via inferior phrenic artery: a case report

**DOI:** 10.1186/s12876-022-02110-6

**Published:** 2022-02-05

**Authors:** Ji Soo Kim, Hyoung Nam Lee, Woong Hee Lee, Suk Hyun Bae

**Affiliations:** 1 Department of Radiology, Soonchunhyang University College of Medicine, Cheonan Hospital, Cheonan, Korea; 2 Department of Radiology, Inje University College of Medicine, Ilsan Paik Hospital, Goyang, Korea

**Keywords:** Embolization, Therapeutic, Hernia, Diaphragmatic, Multidetector Computed Tomography, Carcinoma, Hepatocellular, Case Reports

## Abstract

**Background:**

Transcatheter arterial chemoembolization (TACE) via the inferior phrenic artery has been recognized to have its own therapeutic role without causing serious procedural complications. We report a case of diaphragmatic perforation after repeated TACE sessions conducted via the right inferior phrenic artery.

**Case presentation:**

A 43-year-old man diagnosed with hepatocellular carcinoma was admitted to the hospital with a chief complaint of cough. The patient underwent TACE via the right inferior phrenic artery 3 months prior and was discharged without specific complications. Physical examination revealed decreased breathing sounds in the right lower lung zone. Chest radiograph demonstrated a small right pleural effusion. Chest CT scan revealed a small diaphragmatic perforation. The patient was unable to undergo surgical exploration, and a follow-up CT scan after 2 months revealed progression of the right diaphragmatic perforation with massive herniation of omental fat into the thoracic cavity.

**Conclusions:**

Although TACE via the inferior phrenic artery is a relatively safe procedure, it can be associated with rare but serious complications after repeated procedures. This is a rare case report of diaphragmatic perforation after TACE via the right inferior phrenic artery. Early recognition and prompt surgical management are essential to prevent catastrophic outcomes.

## Background

Transcatheter arterial chemoembolization (TACE) is a standard locoregional treatment for intermediate-stage hepatocellular carcinoma (HCC) [1]. TACE is based on the fact that the liver parenchyma receives a dual blood supply from the hepatic artery and the portal vein, whereas HCCs are supplied exclusively by the hepatic artery [2]. However, HCCs supplied by extrahepatic collateral vessels are frequently encountered in clinical practice with a reported rate of up to 27% in patients undergoing TACE [3]. The inferior phrenic artery is one of the most frequently involved extrahepatic collateral vessels. TACE via the inferior phrenic artery has been recognized to have its own therapeutic role. Potential minor complications include shoulder pain, pleural effusion, basal atelectasis, and transient diaphragmatic weakness [4]. According to previous studies, minor complications occurred in 18–40% of patients after TACE of the inferior phrenic artery [5, 6]. However, irreversible diaphragmatic injury did not develop in any patients. Herein, we report a rare case of diaphragmatic perforation after repeated TACE sessions conducted via the right inferior phrenic artery.

## Case presentation

**Fig. 1:**
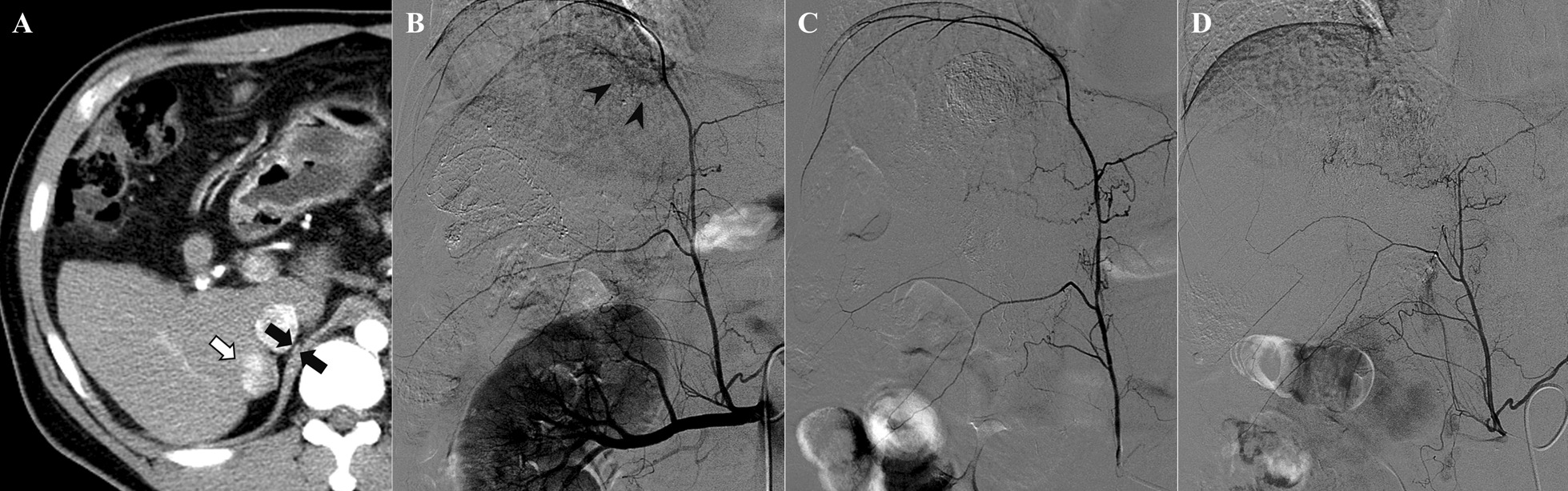
Transcatheter arterial chemoembolization (TACE) via the right inferior phrenic artery. **a** Axial CT scan performed 16 months before the current presentation shows an enhancing tumor (white arrow) in the right hepatic lobe and adjacent inferior phrenic artery (black arrows). **b** Right inferior phrenic angiogram shows hypervascular staining (black arrowheads). Note that the inferior phrenic artery originates in the right renal artery. **c** Selective angiograms after a single session and **d** three sessions of TACE via the right inferior phrenic artery reveal progressive attenuation of the ascending branch

A 43-year-old man diagnosed with multiple HCCs (Barcelona Clinic Liver Cancer stage B), liver cirrhosis and chronic hepatitis B was admitted to the hospital with a chief complaint of cough. The patient underwent three sessions of TACE via the right inferior phrenic artery with emulsion-based formulations using doxorubicin hydrochloride and Lipiodol® (water-in-oil) (Fig. 1) [7]. All procedures were performed by expert interventional radiologists (with at least 5 years of experience) to achieve the greatest technical success while minimizing radiation exposure [8]. For superselective catheterization and embolization, 2.0 F microcatheters (Progreat Alpha; Terumo, Tokyo, Japan) were coaxially used as near as possible to the lesion. No radiofrequency ablation or radiation therapy was performed for disease control.

The treatment response was evaluated with CT (at 1 to 2 months after TACE) according to the modified Response Evaluation Criteria in Solid Tumors by experienced abdominal radiologists (with at least 5 years of experience) [9]. The most recent TACE treatment was conducted 3 months ago and the patient was discharged without specific complications. The initial vital signs were: blood pressure, 147/84 mmHg, heart rate, 73 beats/min, body temperature, 36.6 °C; and respiratory rate, 19 breaths/min. Physical examination revealed decreased breathing sounds in the right lower lung zone. Hematological parameters were: white blood cell count, 4810/µL; hemoglobin, 14.2 g/dL; hematocrit, 39.9%; platelet count, 33,000/µL; aspartate aminotransferase, 149 IU/L; alanine aminotransferase, 41 IU/L; alkaline phosphatase, 202 IU/L; total bilirubin, 1.8 mg/dL; and C-reactive protein, 13.89 mg/L.

**Fig. 2:**
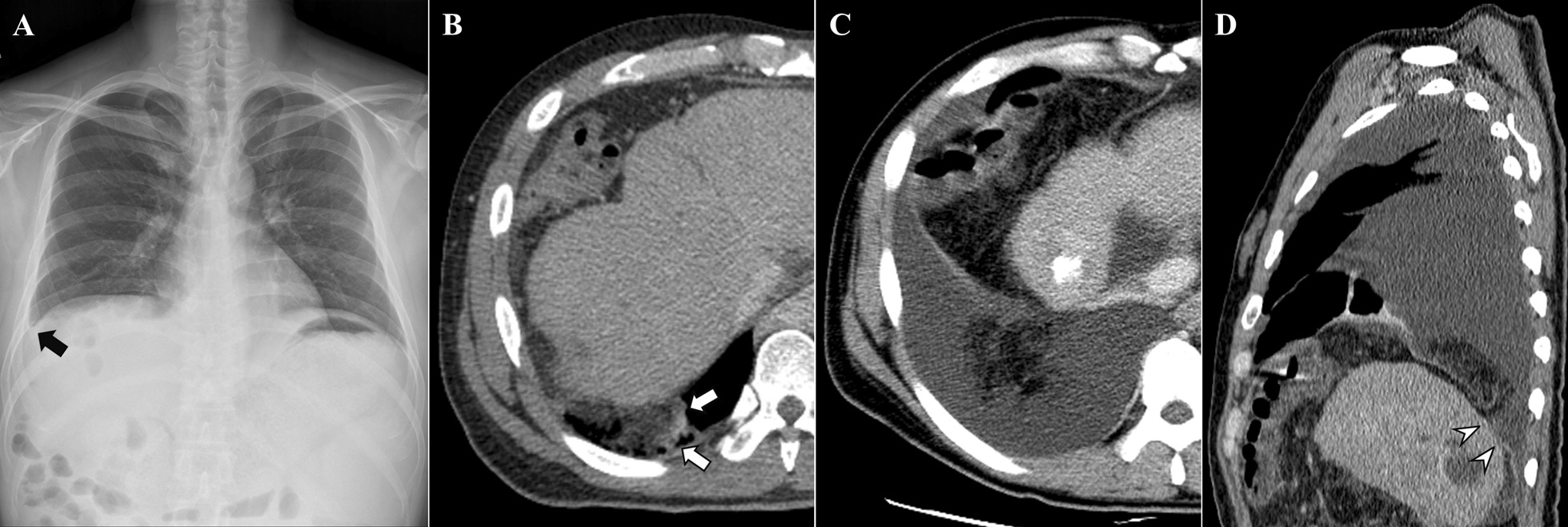
Diaphragmatic perforation and trans-diaphragmatic herniation. **a** Chest radiograph at admission shows mild blunting of the right costophrenic angle (black arrow). **b** Axial CT scan at admission reveals a mushroom-shaped diaphragmatic contour of the herniated omental fat (white arrows). **c** Follow-up axial CT scan after 2 months reveals a large pleural effusion and massive intrathoracic herniation of omental fat without organ entrapment. **d** Reformatted sagittal CT image shows direct discontinuity of the right hemidiaphragm (white arrowheads)

Chest radiograph demonstrated a small right pleural effusion (Fig. 2). A contrast-enhanced CT scan revealed signs of a small diaphragmatic perforation with omental fat herniation. The patient was unable to undergo surgical exploration due to poor general condition and increased risk of morbidity. Cough was exacerbated, and the right pleural effusion was increased, despite the use of diuretics and therapeutic thoracentesis. A follow-up contrast-enhanced CT scan 2 months later revealed progression of the right diaphragmatic perforation, resulting in massive herniation of omental fat into the thoracic cavity (Fig. 2). The patient died of variceal bleeding and disease progression within 6 months.

## Discussion and conclusions

Currently, for intermediate and/or advanced stage HCC, treatment methods include locoregional treatment (e.g., TACE, transarterial radioembolization, bland embolization, cryotherapy, thermal ablation) and systemic therapy (e.g., sorafenib, lenvatinib) [10, 11]. In recent years, the combination therapy of TACE and sorafenib has been increasingly used with promising results [12]. Among them, TACE is the most common treatment option in this clinical setting. Although it is considered a safe procedure, complications can occur, which include hepatic failure, liver abscess, pulmonary embolism, biliary tract injury and acute pancreatitis [13, 14]. Diaphragmatic complications are rare and generally result from TACE via the inferior phrenic artery.

**Fig. 3:**
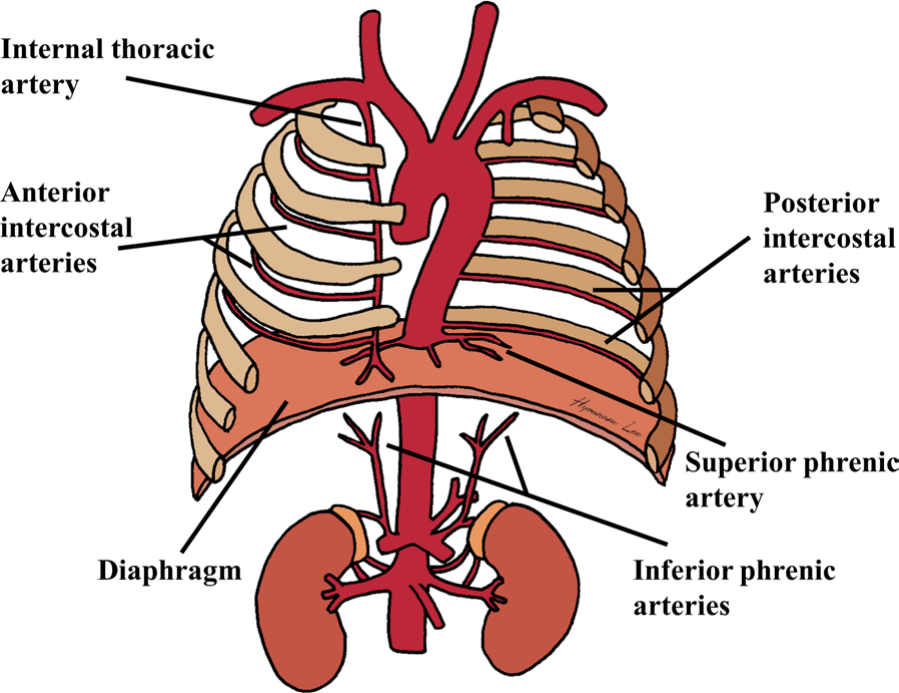
Schematic illustration of the arterial supply to the diaphragm

The diaphragm is vascularized by multiple systemic arteries, including the inferior phrenic, internal thoracic, and lower intercostal arteries (Fig. 3). These arteries anastomose to form arterial circles around the central tendon and the insertion of the diaphragm, and this diversity of blood supply may alleviate ischemic injury during TACE [15]. Inferior phrenic arteries, which are diverse in their origin, including the aorta, celiac axis, or renal artery, are the major source of blood supply to the diaphragm, predominantly the central region. The ascending branches of the bilateral inferior phrenic arteries anastomose with each other, as well as the musculophrenic and pericardiacophrenic branches arising from the internal thoracic artery. The last five intercostal and subcostal arteries supply the costal margins of the diaphragm [16]. The superior phrenic arteries arise from the lower thoracic aorta and supply the superior surface of the diaphragm.

The inferior phrenic artery is the most common extrahepatic collateral vascular supply in HCC [3]. Selective angiography of the right inferior phrenic artery is required if the HCC is located in a bare area and in contact with the right hemidiaphragm [3, 17]. TACE via the inferior phrenic artery has been adopted as a safe and effective treatment [4, 18]. Serious complications are rare but precautions are needed to minimize adverse events. In this context, TACE guided by automated feeder detection software could be a useful option, especially in patients with pre-existing contralateral diaphragmatic dysfunction or underlying pulmonary disease [5, 19, 20]. Diaphragmatic perforation after TACE via the inferior phrenic artery is an extremely rare condition in the literature [5, 6]. Repeated ischemic stress due to multiple treatment sessions and insufficient collateral circulation may explain this fatal complication. In patients requiring repeated procedures via the inferior phrenic artery, switching to systemic therapy may also be considered [11, 21].

Early diagnosis of diaphragmatic perforation following TACE often seems to be difficult due to the low sensitivity and specificity of chest radiographs [22]. Furthermore, minor diaphragmatic damage or perforation with hepatic adherence may be asymptomatic at presentation. Multidetector CT enables better identification of subtle findings suggesting diaphragmatic perforation. As the axial plane is tangential to the dome of the diaphragm, a multiplanar reconstruction technique should be used [23]. CT findings suggestive of diaphragmatic perforation include segmental discontinuity of the diaphragm, the collar sign, and the dependent viscera sign. The collar sign represents intrathoracic herniation of a hollow viscus with or without focal constriction of the viscus at the site of the tear [24]. The “dependent viscera” sign on CT is a sign of diaphragmatic rupture as the herniated viscera lie against the posterior ribs in the thoracic cavity in a dependent position. Focal diaphragmatic thickening and thoracic fluid abutting the abdominal viscera are additional helpful imaging features.

Once diaphragmatic perforation is diagnosed, surgical repair is recommended to avoid fatal delayed complications such as acute intestinal obstruction and perforation. Although rare, the mortality after emergency surgery for strangulated or perforated bowel is high, ranging between 20 and 80%. Even a small defect in the diaphragm is likely to increase in size as a result of the pressure gradient between the abdominal and pleural cavities, which can eventually result in massive herniation [25, 26].

## Conclusion

Although TACE via the inferior phrenic artery is a relatively safe procedure, it can be associated with rare but serious complications after repeated procedures. This is a rare case report of diaphragmatic perforation after TACE via the right inferior phrenic artery. Early recognition and prompt surgical management are essential to prevent catastrophic outcomes.

## Data Availability

Data sharing is not applicable to this article as it is a case report.

## Abbreviations

TACE: Transcatheter arterial chemoembolization
CT: Computed tomography
HCC: Hepatocellular carcinoma
